# Development of ST Elevation Myocardial Infarction and Atrial Fibrillation after an Electrical Injury

**DOI:** 10.1155/2015/953102

**Published:** 2015-01-08

**Authors:** Erdal Gursul, Serdar Bayata, Ercan Aksit, Basak Ugurlu

**Affiliations:** ^1^Biga State Hospital, Kibris Sehitleri Street, Biga, 17200 Canakkale, Turkey; ^2^Katip Celebi University Ataturk Training and Research Hospital, Izmir, Turkey

## Abstract

Electrical energy is a type of energy that is commonly used in daily life. Ventricular premature beats, ventricular tachycardia, ventricular fibrillation, atrial tachycardia, atrial fibrillation, bundle branch blocks, and AV block are arrhythmic complications that are encountered in case of electric shocks. Myocardial infarction is one of the rarely seen complications of electric shocks yet it has fatal outcomes. Coronary arteries were detected to be normal in most of the patients who had myocardial infarction following an electric shock. So, etiology of myocardial infarction is thought to be unrelated to coronary atherosclerosis in these cases. Coronary artery vasospasm is thought to be the primary etiological cause. In our case report, we presented a patient who developed ST elevation MI with atrial fibrillation after an electric shock.

## 1. Introduction

Electrical energy is the commonly used energy type in daily life. However, electric shocks can lead to a wide range of clinical conditions from skin burns to fatal arrhythmic complications. Cardiac effects of electric shock include accelerated hypertension, arrhythmia, acute myocardial infarction (MI), and cardiac rupture [1–3]. Ventricular premature beats, ventricular tachycardia, ventricular fibrillation, atrial tachycardia, atrial fibrillation (AF), bundle branch blocks, and AV block can be seen as the arrhythmic complications of electric shock [3, 4]. MI is one of the rarely seen complications which may lead to fatal outcomes. In most of the cases, patients who had myocardial infarction after electric shock have been reported to have normal coronary arteries. Particularly in case of ST elevation MI, etiology is considered to be vasospasm occurring after electric shock [5].

In our case report, we presented a patient who developed ST elevation MI with atrial fibrillation after an electric shock.

## 2. Case

**Figure 1:**
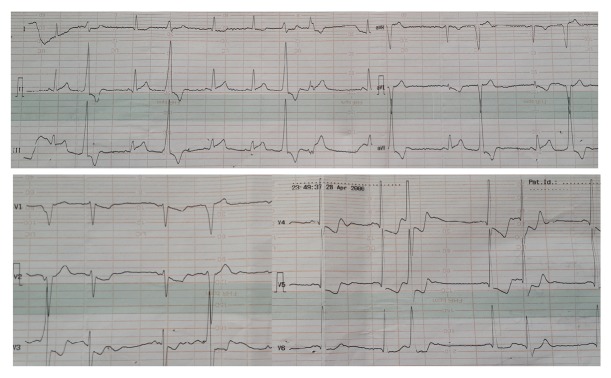
This electrocardiogram demonstrates ventricular premature beats and increased amplitude of the T-wave in inferior derivations.

A 50-year-old male patient admitted to the emergency because of an electric shock which occurred 15 minutes before (alternative current, 50 Hz, 220 V). The patient indicated that as soon as he touched the wall plug to switch on light, he was shocked from his right hand and found himself lying on the floor of his home. He stated that he had lost his consciousness with the shock and after recovery he had found out he had a tightening chest pain radiating to his left arm. His pain had continued increasingly. Arterial blood pressure ratio was measured as 140/80 mmHg on examination of the patient. Pulse was arrhythmic and tachycardic. No lesion was observed regarding the electric shock entry and exit points. Sinus tachycardia, increase in the amplitude of the T-wave at inferior derivations, and ventricular premature beats were monitored in the performed ECG (Figure 1).

**Figure 2:**
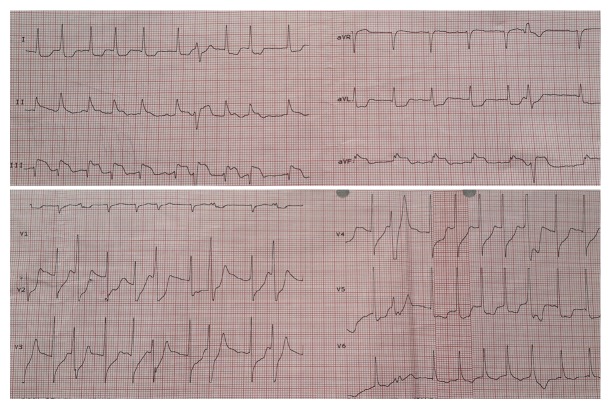
At this electrocardiogram, ST segment elevation in inferior derivations and ST segment depression in V1–V3 are seen with the rhythm of atrial fibrillation.

After establishing a vascular access, routine blood tests were performed and the patient was monitored by connecting to a defibrillator. Nearly after ten minutes, atrial fibrillation developed with a sharpened chest pain. In the performed ECG, ST segment elevation in inferior derivations and ST segment depression in V1–V3 derivations were observed (Figure 2). In order to maintain the ventricular rate under control, intravenous beta-blocker was applied to the patient whose blood pressure was stable. Due to continuous ST segment elevation, ASA 300 mg and clopidogrel 600 mg were given and enoxaparin 0.6 cc was applied subcutaneously. In an effort to exclude a potential Type 1 myocardial infarction, the patient was immediately transferred to a center where primary angioplasty could be performed in 120 minutes. Before coronary angiography in the hospital where he transferred, the patient's chest pain disappeared, ST segment descended to isoelectric line, and the patient returned to sinus rhythm approximately 100 minutes after the detection of symptom. In the performed coronary angiography, normal coronary arteries were determined and dominancy of right coronary artery was observed. The patient was thought to have right coronary arterial vasospasm (Type 2 MI) which was probably triggered by electric shock. A moderate increase in troponin and CK-MB levels was seen in the monitorization of the patient. In echocardiographic examination, EF was estimated as 58% and segmental motion defect was not detected. The patient was discharged with full recovery after four-day-long observation in the hospital.

## 3. Discussion

Electric shock can cause adverse outcomes on human body. Various clinical conditions from simple burn lesions to fatal arrhythmias could be observed in case of electric shocks. In a recently conducted study, electric shock was found to be only 2.2% of the injuries admitted to emergency service; however, it was reported to be 42.9% of the fatal injuries [6]. It is indicated that cardiac pathologies are more frequently encountered when direction of electric current passes through the heart in injuries related to electric shock [7]. Electric shock may lead not only to arrhythmia, but also to myocardial infarction, accelerated hypertension, and myocardial rupture of heart [3, 4]. Pathophysiology of arrhythmia due to electric current has not been fully enlightened yet. Necrotic areas created by current and alterations in cardiac sodium/potassium pump activity are considered to be primary responsible mechanisms [7, 8]. Butler and Gant investigated 182 electric shock incidents over 20 years and they reported only 2 AF cases [2]. Arrowsmith et al. detected 3 ectopic beats and 1 AF (exposed to high voltage) among 145 cases they evaluated regarding cardiac complications over 5 years. In this study, it is emphasized that cardiac complications occurred more frequently if loss of consciousness was seen after electric shock [9]. In our case, loss of consciousness was also developed and cardiac complications were experienced as it is mentioned in literature.

Coronary arteries were detected to be normal in most of the patients who had MI following an electric shock. So, etiology of myocardial infarction is thought to be unrelated to coronary atherosclerosis in these cases. Coronary arterial vasospasm [5], direct thermal effect on myocardium [5], arrhythmia induced hypotension [10], depredation of coronary artery during cardiopulmonary resuscitation [11], and hypoxia due to cardiopulmonary arrest [12] are thought to be primary etiological causes of electric shock induced MI. Development of ST-elevated MI due to electric shock is a rarely encountered situation [13]. It is stated in the literature that right coronary artery lays closer to the chest wall on its course and it is more sensitive to electrical current, so ST elevation in inferior derivations is more frequently observed after electric shock [14]. Al et al. determined normal coronary arteries after electric shock induced inferior MI and they reported that the patient's ECG findings were recovered after 6 hours [15]. Celebi et al. also presented an electric shock induced inferior MI patient with normal coronary arteries. They reported that minimal ST elevation was continued at inferior derivations even after 1 year of shock [16]. In our case, right coronary arterial vasospasm was probably developed after electrical current exposure through right hand. Recovery of ECG findings within 100 minutes after the electric shock and detection of normal coronary arteries in performed angiography also support the idea of vasospasm.

In our case, association of electric shock induced ST elevation at inferior derivations and AF was observed. The AF developed during monitorization can be due to either direct arrhythmic effect of the electric shock or ischemic myocardium. AF disappeared soon after normalization of ST segment. This makes us consider the ischemic etiology on foreground.

Since the primary cause is vasospasm, fibrinolytic therapy should not be considered as the first option for electric shock induced ST elevation MI treatment. Electric shock induced multiple traumas and/or hematomas may also cause contraindication for fibrinolytic treatment. According to all of these, in such cases first treatment option should be percutaneous coronary angioplasty [15, 16].

## 4. Conclusion

Electric shock injuries can result in serious situations such as arrhythmia and myocardial infarction. Arterial vasospasm should be considered as one of the etiologies of ST-elevated MI developed after an electric shock.
